# Students’ COVID-19 vaccine behaviors, intentions, and beliefs at a US Native American-Serving Nontribal Institution (NASNTI)

**DOI:** 10.1186/s13104-023-06439-3

**Published:** 2023-08-18

**Authors:** Tapati Dutta, Jon Agley, Yunyu Xiao, Lilian Golzarri-Arroyo, Sumayyah Ali

**Affiliations:** 1 Public Health Department, Fort Lewis College, 1000 Rim Drive, Schlessman Family Hall 2050, Durango, CO 81301 USA; 2 Prevention Insights, Department of Applied Health Science, School of Public Health Bloomington, Indiana University Bloomington, 809 E. 9th St, Bloomington, IN 47405 USA; 3 Department of Population Health Sciences, Weill Cornell Medical College, 425 E. 61 Street, DV 306, New York, NY 10065 USA; 4 Biostatistics Consulting Center, School of Public Health Bloomington, Indiana University Bloomington, 2719 E. 10th St., Innovation Center Room 224, Bloomington, IN 47405 USA

**Keywords:** COVID-19, Vaccination, Native American, NASNTI, Survey, College, Native American-Serving Nontribal Institution

## Abstract

**Objective:**

Multiple national and international studies of college student COVID-19 vaccination have been recently published, providing important descriptive information and a conceptual basis to inform future decisions about infectious disease prevention in higher education settings. Yet almost no research has examined Native American-Serving Nontribal Institutions (NASNTIs), which occupy a unique space in US higher education in terms of structure and students served. To address that gap, this report describes results from a two-wave cross-sectional survey administered at a NASNTI in Durango, Colorado, as part of a larger study of COVID-19 campus response. Surveys were administered prior to (wave one) and following (wave two) statewide availability of the COVID-19 vaccine for ages 16+. Comparisons between waves used Cramer’s V and Mann-Whitney U tests.

**Results:**

A total of 283 students responded to wave one, and 186 responded to wave two. Notable results included a self-reported COVID-19 vaccination rate (40.1%) at wave one that far exceeded parallel national rates. Injunctive and disjunctive normative beliefs were also less supportive of vaccination among the unvaccinated at wave two compared to wave one. Findings from this study should be considered in the context of all available evidence and not used to make inferences in isolation.

## Introduction

The COVID-19 pandemic introduced a new set of decisions for institutes of higher education, including whether and how to support COVID-19 vaccination uptake among college students. College-centered organizations and initiatives have intensively studied college student vaccination (e.g., the recent *National Survey of College Student COVID-19 Vaccination Uptake, Attitudes, Experiences, and Intention*s by the American College Health Association [1]), and many scholars have examined related factors such as students’ vaccination hesitancy or refusal within the US and internationally [2]. Such work is important both descriptively and in supporting institutes’ responses to similar emergencies in the future.

However, while we have a general sense of college students’ COVID-19 vaccination behaviors, intentions, and beliefs, we might reasonably expect there to be some variation in study findings across different colleges and universities within the US (e.g., there is evidence of differences in students’ personality traits across different colleges, even when accounting for individual demographic differences [3]). Further, there are some types of higher learning institutes in the US for which little COVID-19 vaccination and response information is available. In particular, almost no work has addressed Native American-Serving Nontribal Institutions (NASNTIs), which are institutes of higher education that are not tribal colleges or universities but that serve a student population that is at least 10% Native American [4]. At present, there are approximately 31 NASNTIs in the US [5].

The degree to which general data about college student COVID-19 vaccination behaviors, intentions, and beliefs, might apply to such campuses, is unclear for at least two primary reasons. First, data around Native American vaccine uptake and hesitancy are complex. Kaiser Family Foundation issued a report on April 9, 2021 indicating that American Indian and Alaska Native (AIAN) populations had higher rates of COVID-19 vaccination than other populations, likely attributable to multiple factors including increased availability and discretion for distribution [6]. However, other studies conducted around March 2021 reported lower ‘eagerness’ to get vaccinated for COVID-19 among Native American college students in Nevada [7] and lower COVID-19 vaccine uptake among Native Americans in general, compared to Whites [8]. Rigorous qualitative research found that COVID-19 vaccine attitudes among Native Americans were complex and included multiple culturally specific factors [9]. Second, even beyond those considerations, there are dynamics within the college and tribal environments that may influence health behaviors; for example, NASNTIs may be seeking to center Indigenous values on their campuses [10] in ways that differ from other types of institutions.

This report is part of a series of studies around the COVID-19 response at Fort Lewis College (FLC) in Durango, Colorado (CO), a NASNTI. We share quantitative results from a two-wave cross-sectional study of students’ COVID-19 vaccination behaviors, intentions, and beliefs, conducted (a) before the formal rollout of vaccine eligibility for the college student-age population in CO (April 2, 2021) [11], and then (b) after that rollout. All analyses were exploratory and focused on differences reported by respondents at the two time points across measured variables. We examined vaccination status and beliefs among all respondents and additional intentions and beliefs among the unvaccinated respondents at each time point.

## Methods

The structure of this manuscript was informed by the ‘STROBE Checklist’ for reporting observational studies [12].

### Study design, setting, and participants

This study was a cross-sectional ‘pulse’ survey that was administered during two distinct periods, once from March 9 to 28, 2021, and once from April 14 to 26, 2021. Eligible participants were all students at FLC, a NASNTI located in Durango, CO. Since we were able to solicit responses from all students, the method was technically a census of the full student population [13]. E-mail invitations to participate were sent to all enrolled students from an official campus-level e-mail address in the provost’s office. Each e-mail contained a brief explanation of the study and a link to a survey designed in QualtricsXM. At the end of each survey, participants were able to click a hyperlink to access a separate survey where they could enter their e-mail address to be entered into random drawings for FLC “swag” (e.g., water bottles, sweatshirts). To comply with ethical requirements as outlined by the IRB, e-mail addresses were completely separated from survey responses.

### Variables and measurement

The structure and wording of multiple survey items were drawn from preliminary documentation provided to our team by an author of a national cross-sectional COVID-19 vaccine survey [14]. The following items were used for this study, presented in the order they appeared to participants (complete wording for all sub-items is in Table 2):

- Vaccination (“Vaccines to prevent coronavirus/COVID-19 have been approved by the FDA for use in the United States. The vaccines will be available to different people at different times. Did you already get a vaccine?” [Yes/No]).
  - Vaccinated individuals completed two additional questions around number of doses (not used in this study).
  - Unvaccinated individuals instead completed five questions around their vaccination intentions and beliefs (see next bullet point).
- Theory of Reasoned Action Vaccination Items using the stem, “Assuming a vaccine is available where you live and you are eligible, please indicate how much you agree or disagree with the following statements about making an appointment to be vaccinated within one week.” [1: Strongly disagree, 2: Disagree, 3: Neither agree nor disagree, 4: Agree, 5: Strongly agree] (see Table 2).
- Sociodemographic items (gender, ethnicity, race, and age).
- Attitudes and beliefs around the COVID-19 vaccine such as normative beliefs (e.g., “My making an appointment to be vaccinated within one week is a good thing to do.”) [1: Strongly disagree, 2: Disagree, 3: Neither agree nor disagree, 4: Agree, 5: Strongly agree] (see Table 2).

Six additional questions were asked at the end of the survey but were not used as part of this report due to issues of relevance (general perceptions of FLC) or scope (qualitative responses are part of a separate study).

### Statistical analyses

We used Cramer’s V to compare nominal data between wave one and wave two (vaccination status). For ordinal-by-ordinal comparisons between waves, we treated data as non-repeating, using Mann-Whitney U tests to produce conservative estimates (even though in principle some participants may have responded to both surveys). To compare sociodemographics between waves, we used a *t*-test, including adjustment for unequal variances (age), and chi-square tests (race, gender, and ethnicity). Rather than report significance thresholds, we report exact *p*-values and interpret their plausible meaning [15], including the possibility of spurious results due to multiple pairwise tests (though we note that even the conservative Bonferroni correction would not have changed these specific results).

Missing data were few but did not appear to be missing at random. Instead, they were primarily due to participants who either dropped out following the vaccination status and theory of reasoned action questions (*n* = 8) or who dropped out immediately after responding to the vaccination question (*n* = 8). These early dropouts occurred in both wave 1 (*n* = 11) and wave 2 (*n* = 5) and accounted for almost all cases of missing data. All but two of these early dropouts (*n* = 14) had not been vaccinated at the time of the survey, but of those who were not vaccinated but provided intention data, there was an even mixture of high, low, and moderate intention to get vaccinated within one week. Given this information, we decided to use pairwise exclusion (i.e., each analysis included all available data).

## Results

### Descriptive data

A total of 283 participants took the survey during wave one, and 186 participants took the survey in wave two. According to the FLC Common Data Set, there were 3,443 students enrolled part-time and full-time during the academic year when the survey took place, so wave one captured approximately 8.3% of the population, and wave two captured approximately 5.4% of the population. Table 1 displays gender, ethnicity, and race distributions for each survey wave as well as distributions for the population from the Common Data Set [16]. The mean ages of the samples were 22.3 years for wave one (*n* = 270, *SD* = 5.2) and 23.2 years for wave two (*n* = 179, *SD* = 6.6) (not reported in Table 1). The sample compositions did not significantly differ between waves for age (*t*=-1.58, *df* = 320.5, *95% CI* -2.09, − 0.23, *p* = .115), race (*χ*^*2*^ = 3.73, *p* = .713), gender (*χ*^*2*^ = 0.42, *p* = .811), or ethnicity (*χ*^*2*^ = 0.45, *p* = .505). We did not conduct statistical comparisons between the samples and the Common Data Set but note observationally that our samples had fewer females and more males, as well as more White, non-Hispanic students.

**Table 1:** Descriptive data for samples and population

Wave One Survey (n = 283)		Wave Two Survey (n = 186)		Population*
Variable (*question response* n)	n (%)	Variable (*question response* n)	n (%)	N (%)
**Gender** (n = 274)	-	**Gender** (n = 181)	-	
*Male*	83 (30.3%)	*Male*	60 (33.1%)	1,552 (45.1%)
*Female*	185 (67.5%)	*Female*	117 (64.6%)	1,891 (54.9%)
*Other*	6 (2.2%)	*Other*	4 (2.2%)	-
**Hispanic/Latino** (n = 274)	28 (10.2%)	**Hispanic/Latino** (n = 180)	22 (12.2%)	433 (12.9%)
**Race** (n = 271)	-	**Race** (n = 179)	-	
*American Indian or Alaska * * Native*	82 (30.3%)	*American Indian or Alaska* * Native*	62 (34.6%)	1,103 (32.9%)
*White*	159 (58.7%)	*White*	92 (51.4%)	1,353 (40.4%)
*Asian*	3 (1.1%)	*Asian*	3 (1.7%)	11 (0.3%)
*Black*	1 (0.4%)	*Black*	1 (0.6%)	24 (0.7%)
*More than one race, or * * other race*	23 (8.5%)	*More than one race, or * * other race*	19 (10.7%)	339 (10.1%)

*Population-level metrics combine graduate and undergraduate enrollment for gender (N = 3,443) but only have undergraduate data for race/ethnicity (N = 3,349)

## Outcome data and main results

Outcome data are provided in Table 2, along with statistical comparisons. Approximately 40.1% of the respondents (*n* = 113) reported having been vaccinated (at least one dose) for COVID-19 at wave one, and 66.7% (*n* = 124) reported having been vaccinated (at least one dose) at wave two (*V* = 0.260, *p* < .001). In wave one data, 73.2% of the 82 AIAN respondents reported receiving at least one dose, though 24.5% of the 159 White respondents did as well, a rate still exceeding the US rates for that period [17].

The degree to which the samples reported being worried about contracting COVID-19 was lower in wave two than in wave one (*Z*=-3.876, *p* < .001), whereas the degrees to which they reported having all the information they need about getting vaccinated (*Z*=-3.325, *p* < .001), and knowing when the vaccine will be available to them (*Z*=-6.610, *p* < .001) were higher in wave two than in wave one.

Among subsamples of those who reported not being vaccinated for COVID-19, participants in wave two reported less agreement than those in wave one with four statements: “I will make an appointment to be vaccinated within one week” (*Z*=-4.884, *p* < .001), “My making an appointment to be vaccinated within one week is a good thing to do” (*Z*=-5.578, *p* < .001), “Most people important to me think I should make an appointment to be vaccinated within one week” (*Z*=-4.192, p < .001), and “Most people like me will make an appointment to be vaccinated within one week” (*Z*=-4.104, p < .001).

**Table 2:** Outcome data and statistical comparisons, wave one and wave two

Wave One Survey (n = 283)		Wave Two Survey (n = 186)		Comparison
Variable (*question response* n)	n (%)	Variable (*question response* n)	n (%)	Test Statistic (*p*)
**Vaccination** (n = 282)	-	**Vaccination** (n = 186)	-	V = 0.260 (< 0.001)*
*Yes*	113 (40.1%)	*Yes*	124 (66.7%)	-
*No*	169 (59.9%)	*No*	62 (33.3%)	-
**Variable (*question response *** **n)**	Mean (SD)**	**Variable (*question response *** **n)**	Mean (SD)**	**Test Statistic (** ***p*** )***
I believe the vaccine is effective (n = 272)	4.05 (0.97)	I believe the vaccine is effective (n = 181)	3.92 (1.12)	Z = -0.836 (0.403)
I believe the vaccine is safe (n = 271)	3.92 (1.02)	I believe the vaccine is safe (n = 181)	3.77 (1.17)	Z = -1.003 (0.316)
I will continue to wear a mask and practice hand hygiene behavior after getting vaccinated (n = 270)	4.41 (0.98)	I will continue to wear a mask and practice hand hygiene behavior after getting vaccinated (n = 181)	4.23 (1.14)	Z = -1.593 (0.111)
I will continue to practice physical distancing after getting vaccinated (n = 270)	4.19 (1.07)	I will continue to practice physical distancing after getting vaccinated (n = 181)	4.05 (1.18)	Z = -1.244 (0.213)
I am worried about contracting COVID (n = 272)	3.31 (1.31)	I am worried about contracting COVID (n = 181)	2.82 (1.30)	Z = -3.876 (< 0.001)
I have all the information I need about getting vaccinated (n = 268)	3.48 (1.20)	I have all the information I need about getting vaccinated (n = 181)	3.81 (1.28)	Z = -3.325 (< 0.001)
I know when the vaccine will be available to me (n = 267)	3.23 (1.45)	I know when the vaccine will be available to me (n = 180)	4.18 (0.95)	Z = -6.610 (< 0.001)
**Unvaccinated Wave One (n = 169)**		**Unvaccinated Wave Two (n = 62)**		**Comparison**
**Variable (*question response *** **n)**	Mean (SD)**	**Variable (*question response *** **n)**	Mean (SD)**	**Test Statistic (** ***p*** **)**
I will make an appointment to be vaccinated within one week (n = 165)	3.51 (1.48)	I will make an appointment to be vaccinated within one week (n = 58)	2.31 (1.49)	Z = -4.884 (< 0.001)
My making an appointment to be vaccinated within one week is a good thing to do (n = 165)	3.90 (1.29)	My making an appointment to be vaccinated within one week is a good thing to do (n = 58)	2.69 (1.40)	Z = -5.578 (< 0.001)
Most people important to me think I should make an appointment to be vaccinated within one week (n = 164)	3.57 (1.33)	Most people important to me think I should make an appointment to be vaccinated within one week (n = 58)	2.64 (1.41)	Z = -4.192 (< 0.001)
Most people like me will make an appointment to be vaccinated within one week (n = 164)	3.53 (1.16)	Most people like me will make an appointment to be vaccinated within one week (n = 58)	2.71 (1.30)	Z = -4.104 (< 0.001)
I am confident I can make an appointment to be vaccinated within one week (n = 165)	3.25 (1.41)	I am confident I can make an appointment to be vaccinated within one week (n = 58)	2.91 (1.38)	Z = -1.575 (0.115)

*Comparison using Cramer’s V

**Response options used a Likert-type scale ranging from 1 (Strongly Disagree) to 5 (Strongly Agree)

***Statistical comparisons used Mann-Whitney U tests

## Discussion

This exploratory paper reports COVID-19 vaccination behaviors, intentions, and beliefs before and after formal statewide availability to college-age students. We highlight two areas of particular interest in our findings.

First, the vaccination rate reported at wave one was high relative to national data for the 18-24-year-old age group. Wave one data (March 9 to 28, 2021) for FLC suggested that 40.1% of respondents had received at least one dose *before statewide facilitated access*, with 73.2% of AIAN respondents having received at least one dose. As noted, this was substantially higher than the mean US rates for ages 18 to 24 during this period (March 7, 7.3%; March 27, 14.6%; 2021 [17]). Thus, our findings appear to correspond more closely with the Kaiser Family Foundation report [6] than with research suggesting reduced eagerness or uptake among Native Americans during early 2021.

In interviews with FLC campus leadership, we learned of partnerships between the FLC campus and local tribal entities that were believed to have expedited vaccination rates among students at the college, including those who were not AIAN [18]. Thus, it appears plausible that FLC was able to coordinate with tribal authorities to conjointly facilitate vaccination at the *campus level*; further exploration of such partnerships among NASNTIs might be valuable and informative.

Second, the levels of agreement with injunctive norms (e.g., “perceptions of what most people think should be done”), descriptive norms (e.g., “perceptions of what most people do”) [19], and intention to get vaccinated in the near term (one week), were lower among unvaccinated respondents in wave two compared to wave one. There is evidence from other studies that norms are independently associated with hesitancy to get vaccinated for COVID-19 [20] as well as with the perceived importance of the flu and COVID-19 vaccines (the latter study was conducted prior to COVID-19 vaccine completion) [21]. Our data suggest, but do not confirm, that some students who were vaccinated between waves one and two were those whose normative beliefs were more supportive of getting vaccinated. However, we know that many other factors also are associated with COVID-19 vaccination intentions for adults [22, 23].

This study contributes to our understanding of student COVID-19 vaccination behaviors, intentions, and beliefs at a NASNTI, most interestingly by providing data to support other qualitative claims [18] that a campus-community partnership may have expedited student vaccination rates in spring 2021. Further, at an institutional level, campus leadership was able to incorporate these data into COVID-19 management processes in real time, for example, by adding information about the current vaccination rate to student-facing communication by the Human Resources and Student Engagement office.

### Limitations

We note several limitations to our study. First, the response rates were low (8.3% wave one, 5.4% wave two). While this may have affected generalizability to the FLC student body, Fosnacht et al. recently identified that low response rates (5–10%) can still produce reliable estimates for many measures among students as long as the sampling frame is at least 500 students [24]. This risk was also mitigated by our use of a census (e.g., not subject to sampling bias). Second, these data were not longitudinal but rather cross-sectional at two time points, so our comparisons pertain to proportional means but do not suggest causality in any way. Third, this study did not assess some variables that may be interesting to address in future studies, such as personality attributes. Fourth, it is possible that some students took the survey at both points in time, but due to IRB requirements, we were not able to link identifiers to the survey responses. We therefore analyzed the data as unpaired, which provided less statistical power. As a result, some small differences that were non-significant in our study might have been significant in a counterfactual case where many people took the survey during both waves and data were analyzed as paired. Finally, the study population was a single NASNTI (FLC), so while data may be generalizable to other NASNTIs, the degrees and ways in which that is the case are unclear. Thus, this study should be considered in the context of all available evidence and data and not used to make inferences in isolation.

## Data Availability

Raw study data and analytic code are available through the Open Science Framework Storage System (https://osf.io/59r62/?view_only=1ff21b3f21f6427f913a7b663b8c0553). A preliminary version of this study was presented in an oral format by the lead author (TD) at the 2022 (150th ) American Public Health Association Conference, and components of this study also appear in the associated abstract for that conference.

## Abbreviations

AIAN: American Indian and Alaska Native
CO: Colorado
FLC: Fort Lewis College
NASNTI: Native American-Serving Nontribal Institution
